# A Multicenter Comparison of Carbapenem-Nonsusceptible Enterobacterales and Pseudomonas aeruginosa Rates in the US (2016 to 2020): Facility-Reported Rates versus Rates Based on Updated Clinical Laboratory and Standards Institute Breakpoints

**DOI:** 10.1128/spectrum.01158-22

**Published:** 2022-05-31

**Authors:** Vikas Gupta, Kalvin C. Yu, Jason M. Pogue, Janet A. Watts, Cornelius J. Clancy

**Affiliations:** a Becton, Dickinson, and Company, Franklin Lakes, New Jersey, USA; b Department of Clinical Pharmacy, College of Pharmacy, University of Michigan, Ann Arbor, Michigan, USA; c Department of Medicine, University of Pittsburgh, Pittsburgh, Pennsylvania, USA; University of Texas Southwestern Medical Center

**Keywords:** Enterobacterales, *Pseudomonas aeruginosa*, antimicrobial susceptibility, carbapenems, epidemiology, antibiotic resistance, antimicrobial agents, breakpoints, susceptibility testing

## Abstract

Adoption of revised antimicrobial susceptibility breakpoints is often slow, potentially leading to underreporting of antimicrobial resistance. We compared facility-reported rates of carbapenem nonsusceptibility (NS; intermediate or resistant) with NS rates based on current Clinical and Laboratory Standards Institute (CLSI) breakpoints for Enterobacterales or Pseudomonas aeruginosa isolates in ambulatory and inpatient adults in the BD Insights Research Database (US) from 2016 to 2020. Overall, 77.4% (937,926/1,211,845) and 90.6% (2,157,785/2,381,824) of nonduplicate Enterobacterales isolates with facility-reported susceptibility results had MIC data for ertapenem (ETP) and imipenem/meropenem/doripenem (IPM/MEM/DOR), respectively; 86.9% (255,844/294,426) of P. aeruginosa isolates had MIC data for IPM/MEM/DOR. Facility-reported susceptibility and susceptibility based on CLSI criteria resulted in comparable carbapenem susceptibility rates (99.3% versus 99.1% for ETP-susceptible Enterobacterales, 98.9% versus 98.4% for IPM/MEM/DOR-susceptible Enterobacterales, and 84.9% versus 83.3% for IPM/MEM/DOR-susceptible P. aeruginosa). However, compared with CLSI criteria, facilities underreported Enterobacterales- and IPM/MEM/DOR-NS isolates by 18.8% and 26.5%, respectively, and P. aeruginosa IPM/MEM/DOR-NS isolates by 9.8%. Underreporting was observed for both intermediate and resistant isolates. Our data suggest that delayed adoption of revised breakpoints has a small but potentially important impact on reported rates of antimicrobial resistance. Facilities should be aware of local epidemiology, evaluate potential underreporting of resistance, and assess the related clinical impact.

**IMPORTANCE** Clinicians often base antimicrobial therapeutic decisions on laboratory determinations of pathogen susceptibility to an antibiotic based on MIC breakpoints. MIC breakpoints evolve over time based on new information; between 2010 and 2012 the CLSI lowered carbapenem breakpoints for Enterobacterales and Pseudomonas aeruginosa, and these were subsequently adopted by the US Food and Drug Administration. Carbapenems are important therapeutic options for these difficult-to-treat pathogens, so understanding resistance rates is critically important. However, laboratories can be slow to adopt updated breakpoints. We used MIC data to evaluate whether reports received by hospitals for carbapenem susceptibility were consistent with updated CLSI breakpoints. Although overall susceptibility rates were similar between hospital reports and susceptibility based on updated CLSI criteria, the percentages of carbapenem-resistant isolates were significantly underreported by hospital reports. Delayed adoption of MIC breakpoints may impact epidemiological understanding of resistance and contribute to the spread of resistant pathogens.

## INTRODUCTION

Carbapenems are typically considered “last resort” first-line agents for the treatment of serious Gram-negative infections. Carbapenem-resistant Enterobacterales (CRE), carbapenem-resistant Pseudomonas aeruginosa (CRPA), and other carbapenem-resistant bacterial infections are difficult to treat (1, 2) and have been identified as antibiotic resistance threats by US and international health agencies (3, 4). The Centers for Disease Control and Prevention (CDC) lists CRE as an urgent threat pathogen responsible for an estimated 13,100 hospitalizations, 1,100 deaths, and $130 million in attributable costs in the US in 2017 (3). Infections caused by CRPA, the most common carbapenem-resistant pathogen, have an overall in-hospital mortality rate of approximately 15% (5). The management of carbapenem-resistant Gram-negative bacterial infections pathogens is complicated by high rates of cross-resistance to other antibiotics (6, 7).

Antimicrobial susceptibility and resistance are defined by MIC breakpoints, which are proposed based on *in vitro* drug activity, results from animal model experiments, pharmacokinetic-pharmacodynamic considerations, and, if available, clinical data in humans. Breakpoints are often re-set with the emergence of new data or resistance mechanisms, or with changes in drug dosing or formulations. During the years 2010 to 2012, the Clinical and Laboratory Standards Institute (CLSI) lowered carbapenem breakpoints for Enterobacterales and P. aeruginosa (Table 1) (8, 9), based in part on new data from Monte Carlo simulations and mathematical modeling as well as an improved understanding of carbapenem MIC distributions in strains carrying various genetic resistance determinants (10). These remain the current CLSI breakpoints for carbapenems (11).

**TABLE 1:** Previous and current CLSI carbapenem MIC breakpoints (S/I/R in mg/L) for Enterobacterales and P. aeruginosa (9, 11)

Carbapenem	Enterobacterales		P. aeruginosa	
	Pre-2010 breakpoints	Current breakpoints	Pre-2012 breakpoints	Current breakpoints
Ertapenem	≤2/4/≥8	≤0.5/1/≥2	NA	NA
Imipenem/meropenem/doripenem	≤4/8/≥16	≤1/2/≥4	≤4/8/≥16	≤2/4/≥8

Revised CLSI breakpoints were recognized by the Food and Drug Administration (FDA) in 2012 (for ertapenem [ETP], imipenem [IPM], and doripenem [DOR]) and 2013 (meropenem [MEM]) (12). However, there is typically a significant delay (range of 1 to 9 years) before revised FDA breakpoints are incorporated into commercial antimicrobial susceptibility testing (cAST) systems (8), as there is currently no regulatory mandate concerning updating of revised breakpoints. Manufacturers of cAST systems are required to submit test performance data and obtain clearance with the revised breakpoints prior to approval and widespread use (8). Laboratories may circumvent delays by adopting current CLSI breakpoints based on internal validation of the cAST, but this process is fraught with potential difficulties and considered “off label” (8). Once approved, additional time is required to allow panels and software with the new breakpoints to reach facility laboratories. Given the multiple steps required to update breakpoints on cAST systems, there is usually a significant time lag between CLSI publication of a new breakpoint and its incorporation into reports available to clinicians.

It is therefore perhaps not surprising that a 2019 survey of 982 clinical laboratories in the US found that 372 (37.9%) were not using current CLSI meropenem breakpoints (13). This finding is consistent with a 2015 to 2016 survey of California acute care and long-term care hospitals, which found that only 72% were using current CLSI carbapenem breakpoints and that the time to adoption of these breakpoints was a median of 55 months (14). A public health intervention directed at facilities that continued to use obsolete breakpoints in 2017 resulted in adoption of current carbapenem breakpoints at 47% (16/34) of laboratories after 1 year of follow-up. Outdated breakpoints continued to be used by more than half of the facilities due to a variety of barriers, including lack of awareness and assumptions that manufacturer diagnostics automatically adjust to new guidance (15). These delays in adopting breakpoints can have important consequences in allowing the spread of carbapenem-resistant pathogens. A 2016 study estimated that the 32-month delay between publication and adoption of CLSI carbapenem breakpoints in Orange County, California resulted in 1,821 additional CRE carriers (16).

To better understand the potential impact of delayed adoption of carbapenem breakpoints on under-recognition of carbapenem-resistant pathogens, we compared facility-reported carbapenem-susceptible (S) and carbapenem-nonsusceptible (NS; intermediate [I] or resistant [R]) rates for Enterobacterales and P. aeruginosa to carbapenem-S and -NS rates derived by application of current CLSI MIC breakpoints to MIC data derived from a large group of isolates evaluated throughout the US between 2016 and 2020.

## RESULTS

A total of 326 facilities contributed data to this study; 109 (33.4%) were teaching hospitals and 213 (65.3%) were located in urban locations. One hundred three facilities (31.6%) had <100 beds, 144 (44.2%) had 100–300 beds, and 79 (24.2%) had >300 beds. The geographic regions with the most hospitals were West South Central (59; 18.1%) and Mid Atlantic (56; 17.2%), whereas the fewest hospitals were in New England (5; 1.5%) and the Mountain (12; 3.7%) regions. Given the differences in Enterobacterales CLSI breakpoints for various carbapenems and the lack of an ETP breakpoint for P. aeruginosa, analyses were separated into three groups: (i) Enterobacterales ETP, (ii) Enterobacterales IPM/MEM/DOR, and(iii) P. aeruginosa. Results of MIC interpretations reported by the facility were compared with susceptibility results derived by applying current CLSI MIC breakpoints (Table 1) (9, 11), overall and by hospital demographics (teaching/nonteaching, urban/rural, bed size) and location (US Census Region).

### Comparison of carbapenem susceptibility assessments in Enterobacterales isolates.

**FIG 1:**
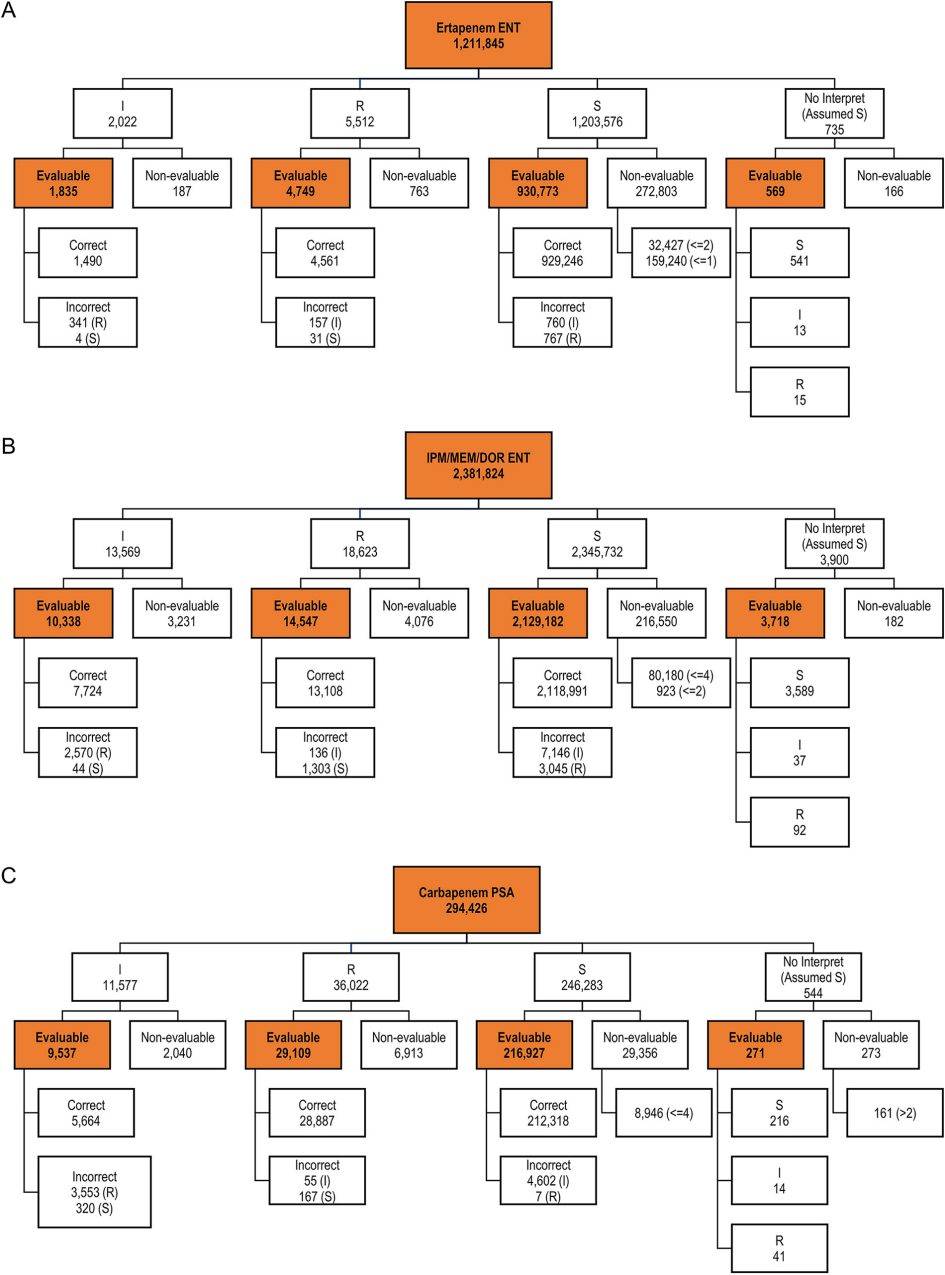
Carbapenem S/I/R evaluations for (A) ertapenem in Enterobacterales (ENT) (B) imipenem/meropenem/doripenem (IPM/MEM/DOR) in ENT, and (C) IPM/MEM/DOR in P. aeruginosa (PSA). “Evaluable” refers to isolates with MIC data available.

Among nonduplicate Enterobacterales isolates with facility-reported susceptibility results, 937,926/1,211,845 (77.4%) also had interpretable MIC results for ETP and 90.6% (2,157,785/2,381,824) had interpretable MIC results for IPM/MEM/DOR (Fig. 1A and B). Susceptibility rates were similar for facility-reported and CLSI breakpoint assessments. ETP-S rates were 99.3% and 99.1% as reported by facilities and using CLSI criteria, respectively, and IPM/MEM/DOR-S rates were 98.9% and 98.4% by facility reporting and CLSI criteria, respectively (Table 2).

**TABLE 2:** Comparison of susceptibility results for facility-reported and CLSI carbapenem breakpoints in evaluable isolates of Enterobacterales and P. aeruginosa^*a*^

Pathogen and interpretation	Ertapenem			Imipenem/meropenem/doripenem		
	Facility-reported	Revised per CLSI breakpoints	Facility versus CLSI	Facility-reported	Revised per CLSI breakpoints	Facility versus CLSI
Enterobacterales (N = 937,926 for ertapenem and 2,157,785 for imipenem/meropenem/doripenem)						
I	1,835 (0.20%)	2,420 (0.26%)	↓ by 24.2%	10,338 (0.48%)	15,043 (0.70%)	↓ by 31.3%
R	4,749 (0.51%)	5,684 (0.61%)	↓ by 16.4%	14,547 (0.67%)	18,815 (0.87%)	↓ by 22.7%
NS (I + R)	6,584 (0.71%)	8,104 (0.86%)	↓ by 18.8%	24,885 (1.15%)	33,858 (1.57%)	↓ by 26.5%
S	931,342 (99.30%)	929,822 (99.13)		2,132,900 (98.85%)	2,123,927 (98.43%)	
P. aeruginosa (N = 255,844)						
I	Not applicable			9,537 (3.7%)	10,335 (4.0%)	↓ by 7.7%
R				29,109 (11.4%)	32,488 (12.7%)	↓ by 10.4%
NS (I + R)				38,646 (15.1%)	42,823 (16.7%)	↓ by 9.8%
S				217,198 (84.9%)	213,021 (83.3%)	

a Data are presented as n (%). Enterobacterales isolates with an interpretation of R met the CDC’s criteria for CRE (an Enterobacterales isolate from a sterile site with an antimicrobial susceptibility test result of R) (3). Calculations of underreporting were determined by subtraction of facility-reported NS isolates from NS isolates as determined by current CLSI criteria.

Systematic application of CLSI breakpoints showed that facility laboratories underreported ETP-I and –R isolates by 24.2% and 16.4%, respectively (*P* < 0.001 for difference between resistance rates determined by CLSI versus facility breakpoints). IPM/MEM/DOR-I and –R isolates were underreported to a larger extent (31.3% and 22.7%, respectively; *P* < 0.001) (Table 2). In total, 1,520/8,104 (18.8%) Enterobacterales identified as ETP-NS by current CLSI breakpoints and 8,973/33,858 (26.5%) of IPM/MEM/DOR-NS isolates were not identified by facility susceptibility reports (Table 2).

### Comparisons of carbapenem susceptibility assessments in P. aeruginosa isolates.

Overall, 86.9% (255,844/294,426) of non-duplicate P. aeruginosa isolates with facility-reported IPM/MEM/DOR susceptibility interpretations also had interpretable MIC results (Fig. 1C). Susceptibility rates were 84.9% and 83.3% as reported by facilities and as determined using CLSI criteria, respectively (Table 2). Facilities underreported carbapenem-NS by 7.7% and 10.4% for I and R isolates, respectively (*P* < 0.001) (Table 2). In total, 4,177/42,823 (9.8%) of carbapenem-NS isolates based on current CLSI breakpoints were not identified by facility laboratories.

### Carbapenem-NS underreporting by facility characteristics.

**FIG 2:**
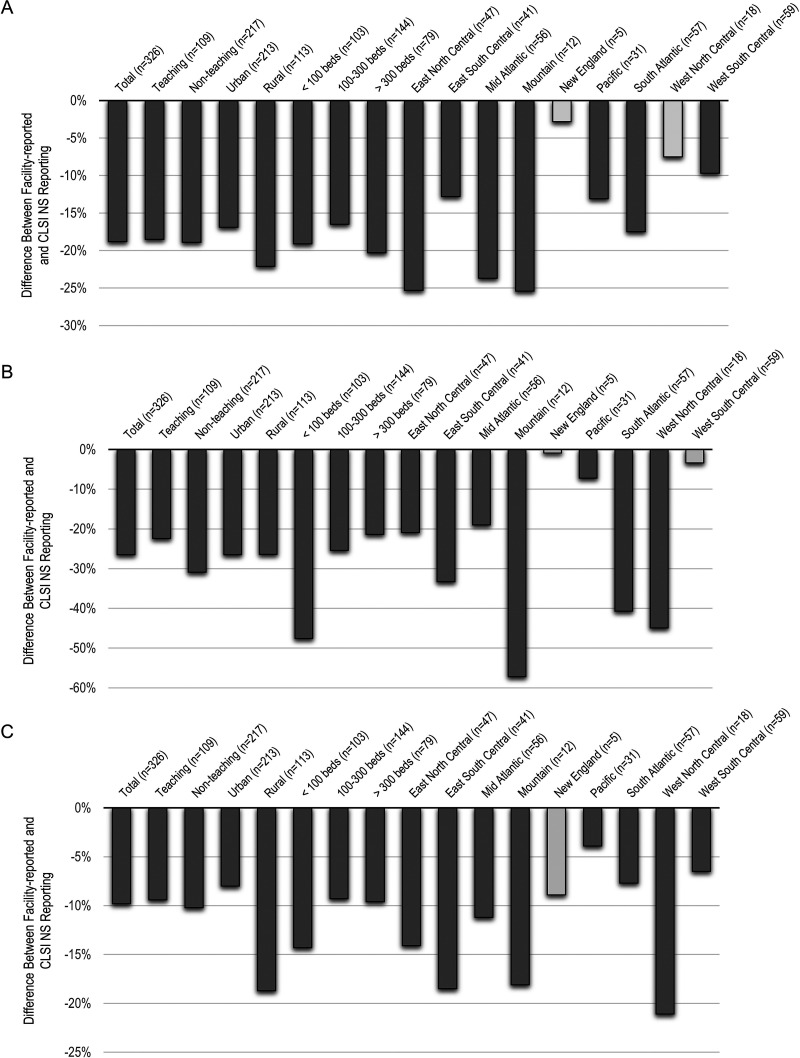
Carbapenem-NS underreporting for facility-reported vs current CLSI breakpoints by hospital demographics. (A) ertapenem-NS Enterobacterales; (B) imipenem/meropenem/doripenem-NS Enterobacterales; (C) imipenem/meropenem/doripenem-NS P. aeruginosa. Darker colored bars indicate *P* < 0.05; n = the number of facilities.

Underreporting of carbapenem-NS Enterobacterales isolates was statistically significant (*P* ≤ 0.018) across all hospital demographics and regions except for New England (*P* = 0.87) and West North Central (*P* = 0.37) for ETP, and New England (*P* = 0.95) and West South Central (*P* = 0.25) for IPM/MEM/DOR (Fig. 2). Similar results were found for carbapenem-NS P. aeruginosa isolates. Facilities in all hospital demographic categories and geographic regions significantly underreported carbapenem-NS rates compared with rates based on current CLSI breakpoints (*P* ≤ 0.030), with the exception of New England (*P* = 0.44).

### Discordances between facility-reported and CLSI carbapenem-NS rates.

**FIG 3:**
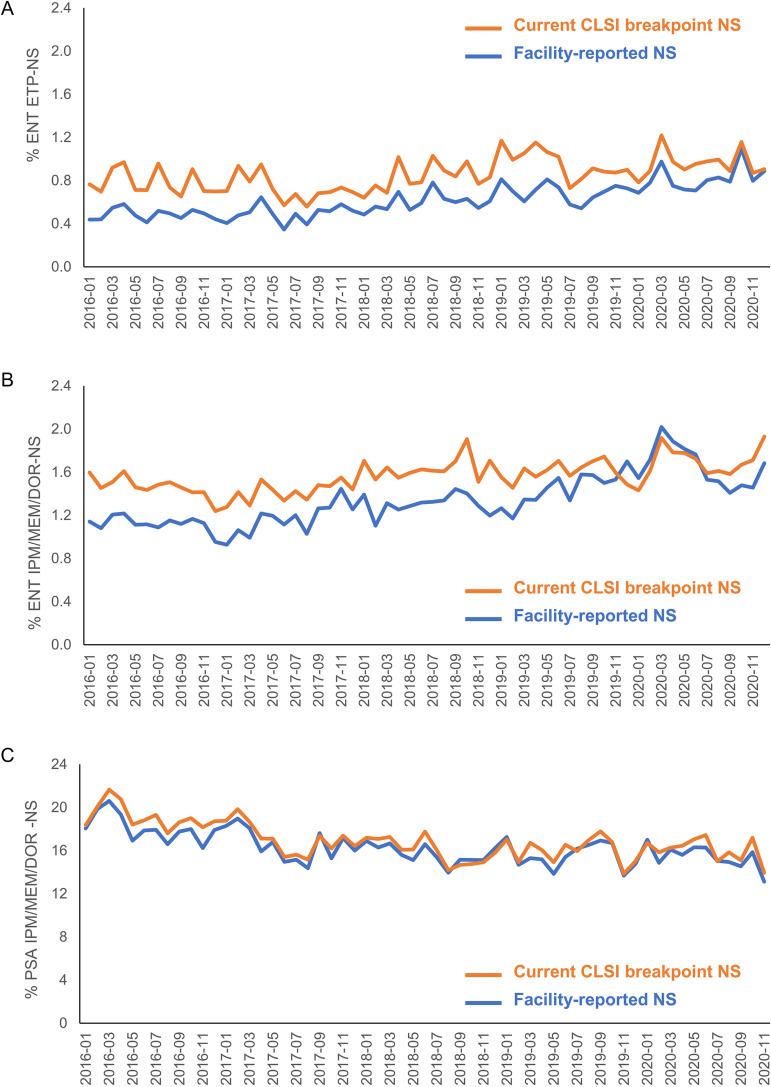
Trends over time for facility-reported and CLSI-revised carbapenem NS rates: (A) ertapenem-NS Enterobacterales (ENT), (B) imipenem/meropenem/doripenem-NS ENT, and (C) imipenem/meropenem/doripenem-NS P. aeruginosa (PSA).

Analyses of carbapenem-NS Enterobacterales and P. aeruginosa showed a divergence between facility-reported rates and rates based on current CLSI breakpoints for most of the time period of the study (2016 to 2020) (Fig. 3). The trend lines appeared to converge toward the end of 2020.

## DISCUSSION

In this retrospective study of over 2 million non-duplicate Enterobacterales isolates and almost 300,000 P. aeruginosa isolates collected from facilities throughout the US between 2016 and 2020, we found that systematic application of current CLSI breakpoints would have had negligible impact on susceptibility rates for these pathogens. However, failure to apply current CLSI breakpoints led to significant underreporting of carbapenem-NS isolates by facilities. Facility laboratories failed to identify 18.8% of ETP-NS and 26.5% of IPM/MEM/DOR-NS Enterobacterales isolates, and 9.8% of IPM/MEM/DOR-NS P. aeruginosa isolates. Assuming that IPM/MEM/DOR-NS Enterobacterales isolates are also ETP-NS, at least 8,973 carbapenem-NS Enterobacterales isolates, and 4,177 carbapenem-NS P. aeruginosa isolates were misidentified as susceptible. Almost half of these misidentified Enterobacterales (4,268 [47.6%]) were carbapenem-R, in line with the CDC definition of CRE (3), and more than three-quarters of misidentified P. aeruginosa (3,379 [80.9%]) were carbapenem-R. Given that the hospitals included in this study account for approximately 10% of admissions in the US (3), our data suggest a sizeable underreporting of carbapenem resistance in the United States. Clinicians should be aware of their local epidemiology, decide if underreporting of carbapenem-NS isolates might be an issue, and assess potential impacts on patients and the community. Although the differences we noted with application of facility versus CLSI breakpoints are modest at the epidemiological level, accurate laboratory interpretative results may be critical to appropriate antibiotic selection in specific patients and to reducing the spread of resistant pathogens.

Research is urgently needed to explore the potential impact of changes in susceptibility classifications on clinical outcomes. Currently, there are limited clinical data validating the recent MIC breakpoint changes. A study of 71 patients with bloodstream infections caused by Gram-negative bacteria (predominantly extended-spectrum beta-lactamase-producing Enterobacterales with a few cases due to P. aeruginosa or Acinetobacter baumannii) who were treated with MER or IPM reported better outcomes in patients whose isolates had an IPM MIC ≤ 2 mg/L compared with those whose isolates had MICs ≥4 mg/L (10). Another study found that higher Enterobacterales carbapenem MICs were associated with longer hospital lengths of stay in patients treated with carbapenems (17). In a recent patient-level analysis of published data that included the two previously described studies as well as additional studies involving Klebsiella pneumoniae (2 studies) and Escherichia coli (1 study), the odds of mortality increased with each increasing MER MIC dilution (OR = 1.51, 95% CI 1.06 to 2.15) (18). Although these studies were small and their interpretation may have been influenced by confounding factors present in retrospective observational studies, the findings combined with our current data suggest that these differences in reported breakpoints may indeed have clinical consequences. Data are needed to determine if dose optimization strategies of MER, such as higher doses or administration via extended or continuous infusions, can be utilized to treat these low-level resistant isolates, or whether novel agents should be preferentially used in the management of these infections.

Accurate identification of CRE and CRPA is an essential first step for infection prevention, and the level of underreporting observed in our study has the potential to impact spread of carbapenem resistance. CRE transmission within, between, and outside medical facilities has been documented (19–21). These trends may have been exacerbated by the recent COVID-19 pandemic, which has affected compliance with traditional infection prevention tenets (22). The effectiveness of efforts to avert the spread of carbapenem-resistant pathogens through initiatives such as the CDC CRE toolkit (23) will be diminished if resistant isolates are not accurately identified by current facility detection systems.

Our analyses suggest that the discrepancy between facility-reported carbapenem-NS rates and NS rates based on current CLSI breakpoints may have decreased between 2016 and 2020. This result may be due to the gradual adoption of CLSI breakpoints at remaining noncompliant facilities. It is also possible that other factors may have led to changes in epidemiology of carbapenem-resistant infections. Additional data will be required to evaluate whether earlier discrepancies in carbapenem-NS rates are now largely resolved, or whether they will reappear in future years. Importantly, the College of American Pathologists Accreditation Programs microbiology checklist was recently updated to require laboratories to use current breakpoints to interpret MIC results by January 1, 2024, and to implement new breakpoints within 3 years of the official publication of the updated breakpoint (24). Requirements such as these may help accelerate the adoption of new breakpoints.

Our study has several limitations. Although we used an algorithm to remove colonizing bacteria from the analyses (25), it is possible that some isolates were colonizers rather than causes of invasive infections. The study was not designed to evaluate whether underreporting of carbapenem-NS Gram-negative pathogens impacted patient outcomes. Certain geographic regions, including New England, had low representation in our database, which may have affected conclusions related to facilities in those areas. Finally, our analyses were based solely on MICs and carbapenemase production was not evaluated. Without access to isolates, we were unable to determine if specific resistance mechanisms or genetic features were overrepresented among isolates that misidentified as carbapenem-S or to evaluate the contribution of facility-specific “expert” rules to the categorization of these isolates.

In conclusion, we found that approximately 10% to 25% of carbapenem-NS Enterobacterales and P. aeruginosa based on current CLSI breakpoints were misidentified as carbapenem-S by US health care facility laboratories from 2016 to 2020. Clinicians should be aware of the potential for underreporting of carbapenem resistance. As new antibiotics and breakpoints are released in the future, it will be important to monitor the accuracy and clinical impact of susceptibility and resistance reporting.

## MATERIALS AND METHODS

### Study design.

This was a retrospective descriptive cohort study of antimicrobial susceptibility rates based on data from US ambulatory and inpatient settings in the BD Insights Research Database (Becton, Dickinson, and Company, Franklin Lake, NJ). The database captures approximately 4.5 million of the estimated 34.5 million annual hospital admissions in the US (3) and provides diverse demographic and geographic representation across the country (26–29). The study was performed in accordance with all relevant guidelines and regulations, including the Declaration of Helsinki. Outcome studies using this retrospective, deidentified data set were approved and informed consent was waived by the New England Institutional Review Board (Wellesley, Massachusetts; No. 120180023).

### Pathogen antimicrobial susceptibility assessments.

For S/NS assessments, we evaluated all isolates with appropriate antimicrobial susceptibility data collected between the first quarter of 2016 and the fourth quarter of 2020 from adults with a positive Enterobacterales or P. aeruginosa culture (first isolate per 30-day period from blood, respiratory, urine, skin/wound, intra-abdominal, or other sources). Results likely to be associated with colonization (e.g., environmental/surveillance specimens such as rectal or nasal swabs) or contamination (dermatology samples and urine isolates with fewer than 10,000 colonies per mL urine) were excluded by use of a previously described algorithm (25). Facility-reported carbapenem susceptibility results were based on institutional laboratory information system feed designations of S or NS (defined as I or R) as determined by commercial or local panels for Enterobacterales (Escherichia coli, Klebsiella pneumoniae, Klebsiella oxytoca, Klebsiella aerogenes, Proteus mirabilis, Enterobacter cloacae, Serratia marcescens, Citrobacter freundii, Providencia stuartii, and Morganella morganii) and P. aeruginosa.

For analyses of the effect of 2021 CLSI MIC breakpoints on S/NS assessments, we evaluated all Enterobacterales and P. aeruginosa isolates with appropriate reported MICs. Those without MICs listed or for which the lowest MIC given was above the new CLSI breakpoint (e.g., ≤4) were considered non-evaluable and were excluded from analysis (Fig. 1). Current CLSI MIC breakpoints for ETP, IPM, MEM, and DOR are shown in Table 1 (9, 11). Because ETP has different Enterobacterales breakpoints than IPM/MEM/DOR and isolates can be NS to ETP while susceptibility is retained to IMP/MEM/DOR, analyses of ETP-NS and IPM/MEM/DOR-NS were conducted separately. If isolates were tested against more than one of the three carbapenems included in combined analyses (IPM/MEM/DOR), the highest interpretive category was used as the facility-reported result. Each isolate was only included once in a given combined analysis, even if it had susceptibility results for multiple carbapenems.

### Statistical analysis.

Descriptive data are provided for S/I/R. Two-proportion Z tests were used to assess the difference between facility-reported S/I/R results and S/I/R results based on CLSI criteria overall and by hospital demographics(teaching/nonteaching, urban/rural, bed size) and location (US Census Region). *P*-values <0.05 indicated a significant difference.

## References

[1] Thaden JT, Pogue JM, Kaye KS. 2017. Role of newer and re-emerging older agents in the treatment of infections caused by carbapenem-resistant Enterobacteriaceae. Virulence 8:403–416. doi:10.1080/21505594.2016.1207834. 27384881 PMC5477716

[2] Tamma PD, Aitken SL, Bonomo RA, Mathers AJ, van Duin D, Clancy CJ. 2021. Infectious Diseases Society of America Guidance on the treatment of extended-spectrum beta-lactamase producing Enterobacterales (ESBL-E), carbapenem-resistant Enterobacterales (CRE), and *Pseudomonas aeruginosa* with difficult-to-treat resistance (DTR-*P aeruginosa*). Clin Infect Dis 72:1109–1116. doi:10.1093/cid/ciaa1478. 33830222

[3] Centers for Disease Control and Prevention. 2019. Antibiotic resistance threats in the United States, 2019. https://www.cdc.gov/drugresistance/pdf/threats-report/2019-ar-threats-report-508.pdf.

[4] Tacconelli E, Carrara E, Savoldi A, Harbarth S, Mendelson M, Monnet DL, Pulcini C, Kahlmeter G, Kluytmans J, Carmeli Y, Ouellette M, Outterson K, Patel J, Cavaleri M, Cox EM, Houchens CR, Grayson ML, Hansen P, Singh N, Theuretzbacher U, Magrini N, WHO Pathogens Priority List Working Group. 2018. Discovery, research, and development of new antibiotics: the WHO priority list of antibiotic-resistant bacteria and tuberculosis. Lancet Infect Dis 18:318–327. doi:10.1016/S1473-3099(17)30753-3. 29276051

[5] Babiker A, Clarke LG, Saul M, Gealey JA, Clancy CJ, Nguyen MH, Shields RK. 2021. Changing epidemiology and decreased mortality associated with carbapenem-resistant Gram-negative bacteria from 2000–2017. Clin Infect Dis 73:e4521–e4530. doi:10.1093/cid/ciaa1464. 32990319 PMC8662792

[6] Buehrle DJ, Shields RK, Clarke LG, Potoski BA, Clancy CJ, Nguyen MH. 2017. Carbapenem-resistant *Pseudomonas aeruginosa* bacteremia: risk factors for mortality and microbiologic treatment failure. Antimicrob Agents Chemother 61:e01243-16. doi:10.1128/AAC.01243-16. 27821456 PMC5192105

[7] Durante-Mangoni E, Andini R, Zampino R. 2019. Management of carbapenem-resistant *Enterobacteriaceae* infections. Clin Microbiol Infect 25:943–950. doi:10.1016/j.cmi.2019.04.013. 31004767

[8] Humphries RM, Abbott AN, Hindler JA. 2019. Understanding and addressing CLSI breakpoint revisions: a primer for clinical laboratories. J Clin Microbiol 57:e00203-19. doi:10.1128/JCM.00203-19. 30971460 PMC6535595

[9] O’Donnell JN, Miglis CM, Lee JY, Tuvell M, Lertharakul T, Scheetz MH. 2016. Carbapenem susceptibility breakpoints, clinical implications with the moving target. Expert Rev anti Infec Ther 14:389–401. doi:10.1586/14787210.2016.1159131. 26918486

[10] Esterly JS, Wagner J, McLaughlin MM, Postelnick MJ, Qi C, Scheetz MH. 2012. Evaluation of clinical outcomes in patients with bloodstream infections due to Gram-negative bacteria according to carbapenem MIC stratification. Antimicrob Agents Chemother 56:4885–4890. doi:10.1128/aac.06365-11. 22777044 PMC3421845

[11] Clinical and Laboratory Standards Institute (CLSI). 2021. Performance standards for antimicrobial susceptibility testing. CLSI document M100. 31st ed Clinical and Laboratory Standards Institute, Wayne, PA. 10.1128/JCM.00213-21 PMC8601225 34550809

[12] Humphries RM, Hindler JA. 2016. Emerging resistance, new antimicrobial agents but no tests! The challenge of antimicrobial susceptibility testing in the current US regulatory landscape. Clin Infect Dis 63:83–88. doi:10.1093/cid/ciw201. 27025822

[13] Simner PJ, Rauch CA, Martin IW, Sullivan KV, Rhoads D, Rolf R, She R, Souers RJ, Wojewoda C, Humphries RM. 2022. Raising the bar: improving antimicrobial resistance detection by clinical laboratories by ensuring use of current breakpoints. Open Forum Infect Dis 9:ofac007. doi:10.1093/ofid/ofac007. 35146049 PMC8826219

[14] Humphries RM, Hindler JA, Epson E, Horwich-Scholefield S, Miller LG, Mendez J, Martinez JB, Sinkowitz J, Sinkowitz D, Hershey C, Marquez P, Bhaurla S, Moran M, Pandes L, Terashita D, McKinnell JA. 2018. Carbapenem-resistant Enterobacteriaceae detection practices in California: what are we missing? Clin Infect Dis 66:1061–1067. doi:10.1093/cid/cix942. 29099915

[15] McKinnell JA, Bhaurla S, Marquez-Sung P, Pucci A, Baron M, Kamali T, Bugante J, Schwartz B, Balter S, Terashita D, Butler-Wu S, Gunzenhauser J, Hindler J, Humphries RM. 2019. Public health efforts can impact adoption of current susceptibility breakpoints, but closer attention from regulatory bodies is needed. J Clin Microbiol 57:e01488-18. doi:10.1128/JCM.01488-18. 30567751 PMC6425187

[16] Bartsch SM, Huang SS, Wong KF, Slayton RB, McKinnell JA, Sahm DF, Kazmierczak K, Mueller LE, Jernigan JA, Lee BY. 2016. Impact of delays between Clinical and Laboratory Standards Institute and Food and Drug Administration revisions of interpretive criteria for carbapenem-resistant *Enterobacteriaceae*. J Clin Microbiol 54:2757–2762. doi:10.1128/JCM.00635-16. 27582516 PMC5078554

[17] Patel TS, Nagel JL. 2015. Clinical outcomes of *Enterobacteriaceae* infections stratified by carbapenem MICs. J Clin Microbiol 53:201–205. doi:10.1128/JCM.03057-14. 25378572 PMC4290923

[18] O'Donnell JN, Rhodes NJ, Biehle LR, Esterly JS, Patel TS, McLaughlin MM, Hirsch EB. 2020. Assessment of mortality stratified by meropenem inhibitory concentration in patients with Enterobacteriaceae bacteraemia: a patient-level analysis of published data. Int J Antimicrob Agents 55:105849. doi:10.1016/j.ijantimicag.2019.11.006. 31770628

[19] Bower CW, Fridkin DW, Wolford HM, Slayton RB, Kubes JN, Jacob JT, Ray SM, Fridkin SK. 2020. Evaluating movement of patients with carbapenem-resistant Enterobacteriaceae infections in the greater Atlanta metropolitan area using social network analysis. Clin Infect Dis 70:75–81. doi:10.1093/cid/ciz154. 30809636

[20] Kelly AM, Mathema B, Larson EL. 2017. Carbapenem-resistant Enterobacteriaceae in the community: a scoping review. Int J Antimicrob Agents 50:127–134. doi:10.1016/j.ijantimicag.2017.03.012. 28647532 PMC5726257

[21] Köck R, Daniels-Haardt I, Becker K, Mellman A, Friedrich AW, Mevius D, Schwarz S, Jurke A. 2018. Carbapenem-resistant *Enterobacteriaceae* in wildlife, food-producing, and companion animals: a systematic review. Clin Microbiol Infect 24:1241–1250. doi:10.1016/j.cmi.2018.04.004. 29654871

[22] Baker MA, Sands KE, Huang SS, Kleinman K, Septimus EJ, Varma N, CDC Prevention Epicenters Program. 2021. The impact of COVID-19 on healthcare-associated infections. Clin Infect Dis doi:10.1093/cid/ciab688. PMC8385925 34370014

[23] Centers for Disease Control and Prevention. Facility guidance for control of carbapenem-resistant *Enterobacteriacae*. November 2015 update – CRE toolkit. https://www.cdc.gov/hai/pdfs/cre/CRE-guidance-508.pdf.

[24] Neff NV. 2021. AST and safety at core of microbiology checklist changes. CAP TODAY. October 2021. https://www.captodayonline.com/ast-and-safety-at-core-of-microbiology-checklist-changes/.

[25] Brossette SE, Hacek DM, Gavin PJ, Kamdar MA, Gadbois KD, Fisher AG, Peterson LR. 2006. A laboratory-based, hospital-wide, electronic marker for nosocomial infection: the future of infection control surveillance? Am J Clin Pathol 125:34–39. doi:10.1309/502AUPR8VE67MBDE. 16482989

[26] McCann E, Srinivasan A, DeRyke CA, Ye G, DePestel DD, Murray J, Gupta V. 2018. Carbapenem-nonsusceptible Gram-negative pathogens in ICU and non-ICU settings in US hospitals in 2017: a multicenter study. Open Forum Infect Dis 5:ofy241. doi:10.1093/ofid/ofy241. 30364442 PMC6194421

[27] Gupta V, Ye G, Olesky M, Lawrence K, Murray J, Yu K. 2019. Trends in resistant Enterobacteriaceae and Acinetobacter species in hospitalized patients in the United States: 2013–2017. BMC Infect Dis 19:742. doi:10.1186/s12879-019-4387-3. 31443635 PMC6708167

[28] Gupta V, Yu KC, Schranz J, Gelone SP. 2021. A multicenter evaluation of the US prevalence and regional variation in macrolide-resistant S*. pneumoniae* in ambulatory and hospitalized adult patients in the US. Open Forum Infect Dis 8:ofab063. ofab063. doi:10.1093/ofid/ofab063. 34250183 PMC8266646

[29] Dunne MW, Puttagunta S, Aronin SI, Brossette S, Murray J, Gupta V. 2022. Impact of empirical antibiotic therapy on outcomes of outpatient urinary tract infection due to nonsusceptible Enterobacterales. Microbiol Spectr 10:e0235921. doi:10.1128/spectrum.02359-21. 35138150 PMC8826825

